# Er:YAG laser-induced damage to a dental composite in simulated clinical scenarios for inadvertent irradiation: an in vitro study

**DOI:** 10.1007/s10103-021-03348-4

**Published:** 2021-07-11

**Authors:** Katharina Kuhn, Carmen U. Schmid, Ralph G. Luthardt, Heike Rudolph, Rolf Diebolder

**Affiliations:** 1 Center of Dentistry, Department of Prosthetic Dentistry, Ulm University, Albert-Einstein-Allee 11, 89081 Ulm, Germany; 2 Center for Dental and Oral Medicine, Department of Orthodontics, University Medical Center Hamburg-Eppendorf, 20246 Hamburg, Germany; 3 Institut für Lasertechnologien in der Medizin und Messtechnik, 89081 Ulm, Germany

**Keywords:** Er:YAG laser, Dental composite, Inadvertent irradiation, Laser-induced damage

## Abstract

Inadvertent Er:YAG laser irradiation occurs in dentistry and may harm restorative materials in teeth. The aim of this in vitro study was to quantify Er:YAG laser-induced damage to a nanohybrid composite in simulated clinical scenarios for inadvertent direct and indirect (reflection) laser irradiation. The simulation was performed by varying the output energy (OE;direct˃indirect) reaching the specimen and the operating distance (OD;direct˂indirect). Composite specimens were irradiated by an Er:YAG laser. The ablation threshold was determined and clinically relevant parameters were applied (*n* = 6 for each OE/OD combination) for direct (OE: 570 mJ/OD: 10 mm, OE: 190 mJ/OD: 10 mm) and indirect irradiation (OE: 466 mJ/OD: 15 mm, OE: 57 mJ/OD: 15 mm, OE: 155 mJ/OD: 15 mm, OE: 19 mJ/OD: 15 mm). The extent of damage in the form of craters was evaluated using a laser scanning microscope (LSM) and a conventional light microscope (LM). The ablation threshold was determined to be 2.6 J/cm^2^. The crater diameter showed the highest value (LM: 1075 ± 18 µm/LSM: 1082 ± 17 µm) for indirect irradiation (reflectant:dental mirror) (OE: 466 mJ/OD: 15 mm). The crater depth showed the highest and comparable value for direct (OE: 570 mJ/OD: 10 mm; LSM: 89 ± 2 µm) and indirect irradiation (OE: 466 mJ/OD: 15 mm; LSM: 90 ± 4 µm). For each OD, the crater diameter, depth, and volume increased with higher laser fluence. However, the OD—and thus the laser spot diameter—also had an enlarging effect. Thus, indirect irradiation (reflectant:dental mirror) with only 47% of the laser fluence of direct irradiation led to a larger diameter and a comparable depth. The three-dimensional extent of the crater was large enough to cause roughening, which may lead to plaque accumulation and encourage caries, gingivitis, and periodontitis under clinical conditions. Clinicians should be aware that reflected irradiation can still create such craters.

## Introduction

Non-target tissues can be damaged by inadvertent laser irradiation during dental laser application, which is repeatedly addressed in publications on laser safety [1–3]. In addition to non-target tissues outside the mouth (e.g., eye), intraoral structures can be affected. These may be adjacent teeth and their restorations. Considering the increase in the use of esthetic restorative materials, such as composites, inadvertent laser irradiation may easily hit composite restorations. In particular, Er:YAG lasers, which are used in many clinical fields in dentistry [4–6], are known to ablate composites [7–12] and cause thermal effects [7, 8]. If irradiation occurs inadvertently, the resulting ablation and thermal effects (e.g., melting) are considered laser-induced damage.

The extent of Er:YAG laser-induced ablation has been investigated for several intraoral structures. Ablation only occurs if the ablation threshold—a material-specific laser fluence—for the specific laser wavelength is exceeded. For dental hard tissues, the range of ablation thresholds for the Er:YAG laser is 3.9–11 J/cm^2^ for enamel [13–15] and 2.7–4 J/cm^2^ for dentin [15, 16]. The values for the ablation thresholds (2.6–4.7 J/cm^2^) for four representatives of the composite material class (Multilink Automix, SpeedCEM, Variolink II, and Variolink Veneer) [17] are in the range of the ablation threshold for dentin. In comparison, conventional dental cements show lower ablation thresholds [8]. However, it should be noted that the ablation threshold varies depending on its determination methodology [15], the specific conditions at the laser [13], and the specific condition of the material itself (e.g., sclerotic dentin shows increased ablation thresholds) [18]. A comparison is therefore only possible to a limited extent. The main mechanism of composite ablation differs from that of dental hard tissue ablation. In the case of Er:YAG laser-induced ablation of enamel and dentin, vaporization of the absorber water occurs, which leads to microexplosive tearing out of dental hard tissue and the formation of craters [19]. This process is only possible if, as here, the vaporization temperature of the absorber is lower than the melting point of the solid matrix containing the absorber [8, 20]. In composites, this condition is not present, and rapid melting occurs upon Er:YAG laser irradiation [8], resulting in large expansion forces due to the volume increase upon melting and thermal vaporization [7, 11]. This also results in explosive ejection of material and thus ablation craters [8, 11] but with evidence of the melting processes that took place [7, 8]. The Er:YAG laser ablation efficiency differs depending on the composition and age of the composites. A hybrid composite could be ablated more easily than a microfilled and condensable composite [11], and aged composites seemed to be more easily ablated [10]. The diameter, depth, and volume of the composite ablation craters increase with increasing laser energy at a constant operating distance—thus a constant laser spot diameter—at first and then seem to reach a saturation value [11].

For the quantification of crater dimensions, a light microscope is usually used [8, 10, 21]. With light microscopy, direct determination of the three-dimensional form of laser-induced damage is not possible. By contrast, a laser scanning microscope (LSM) is suitable for analyzing the three-dimensional form of the crater, including its depth and volume. A study on Er:YAG laser-induced ablation in dentin quantified the depth and volume of craters, but not the diameter, with an LSM [22]. To the best of the authors’ knowledge, no study has quantified Er:YAG laser-induced craters on composites with LSM.

Inadvertent laser irradiation can occur directly to immediately adjacent non-target tissue, especially while working in the non-contact mode or indirectly due to reflection [2, 23]. The reflected laser beam travels a longer distance before hitting the non-target tissue (here, composite) than in the case with inadvertent direct irradiation. This usually results in a larger laser spot diameter and reduced energy due to losses. This clinical scenario for reflection shows that the issue of laser-induced damage due to inadvertent irradiation is inextricably linked to the influence of the energy reaching the composite and the operating distance traveled and therefore the laser spot diameter. The influence of the energy on the crater dimensions at a constant laser spot diameter was investigated as described above. However, the laser spot diameter might be another important parameter.

The scenario for reflection as described above results in a smaller laser fluence. Only if the resulting laser fluence is still above the ablation threshold of the specific composite will ablation occur. Thus, it is unclear whether Er:YAG laser-induced damage in the form of ablation occurs at all and to what degree in different clinical scenarios for inadvertent irradiation and whether thermal damage occurs. To the best of the authors’ knowledge, this issue has not been investigated in the literature, although the problem of laser-induced damage to intraoral structures (here, composite restorations) due to inadvertent laser irradiation is repeatedly addressed [2, 24].

Therefore, the aim of this in vitro study was to detect and quantify Er:YAG laser-induced damage (ablation and thermal effects) on a nanohybrid composite in terms of clinically relevant irradiation parameters that simulated clinical scenarios for inadvertent laser irradiation. The study should give an answer to the question, if damage still occurs with indirect inadvertent Er:YAG laser irradiation when simulating reflection and if so, to what extent. This meant at the same time to study the influence of the output energy and operating distance on the extent of the resulting damage simultaneously. For the analyses, in addition to a laser scanning microscope (LSM), the standard measuring device—a conventional light microscope (LM)—should be used to check the comparability of the results of the methods to each other and thus to other studies.

## Materials and methods

The ablation threshold was determined on nanohybrid composite specimens (Venus Diamond, Heraeus Kulzer, Hanau, Germany) for the Er:YAG laser (KEY Laser 3 + with the universal handpiece 2060, non-contact mode, KaVo Dental GmbH, Biberach, Germany) with a wavelength of 2.94 µm (operating mode, pulsed; pulse duration, 200–700 µs). This dental laser has adjustable energies between 80 and 600 mJ, as well as repetition rates between 2 and 30 Hz. Subsequently, further nanohybrid composite specimens made of the same material were irradiated using the same Er:YAG laser and handpiece with clinically relevant laser parameters varying in operating distance and output energy, which simulated clinical scenarios for inadvertent laser irradiation. Thus, laser damage was induced in the filling material in the form of thermal effects and craters. The extent of laser damage was quantified using a light microscope (Olympus SZX7, Olympus, Tokyo, Japan) to measure the diameter of the craters and using a laser scanning microscope (LSM 510 Axiovert 200 M, Carl Zeiss AG, Oberkochen, Germany) to measure the diameter, depth, and volume of the craters.

### Specimen preparation

**Fig. 1:**
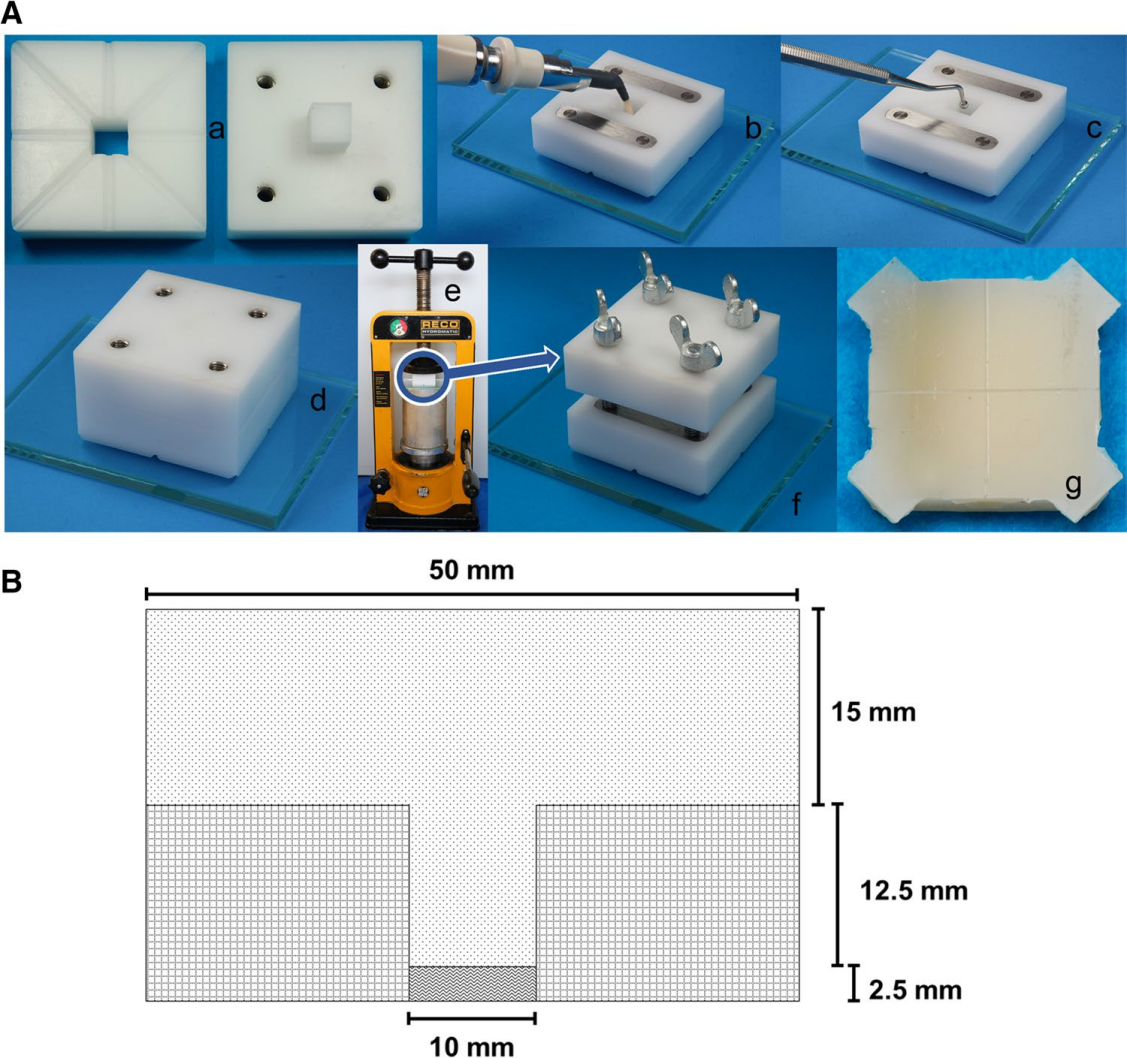
**A** a Cementation device made of polyoxymethylene; left, lower part of the cementation device (bottom side) with a through-hole in the center and drain channels for the composite; right, upper part of the cementation device (bottom side) with a stamp in the center and four through-hole threads with glued-in thread inserts for screws. b Application of composite in the center hole of the lower part of the cementation device (see A) placed on a glass plate (here, upper side with two metallic stripes); the metallic stripes were glued in flush in milled recesses so that they were at the same level as the immediate surroundings. c Compression of composite with a ball-shaped plugger. d Assembled cementation device. e Cementation device on a glass plate in a prosthesis press. f Detail: M5 brass screws in the upper part of the cementation device screwed in against the plane surface of the metallic stripes (see Fig. 1b, metallic stripes with four corresponding circular signs of wear) to separate the upper and lower part of the cementation device. g Flat composite specimen (bottom side) with four squares after scratching a cross and trimming the drain channels. **B** Schematic side view cut through in the middle of the assembled cementation device with dimensions

Flat composite specimens (10 mm × 10 mm × 2.5 mm) were created (Fig. 1A) for both the determination of the ablation threshold and for Er:YAG laser irradiation for laser-induced damage. For this purpose, a custom-made cementation device (Figs. 1A and B) was produced by subtractive methods (milling machine). It was made of polyoxymethylene because of its isolating properties against composites. The cementation device and a glass plate were cleaned with ethanol. The composite was applied (Fig. 1A, b) and inserted (Fig. 1A, c) in the center hole of the lower part of the cementation device standing on a glass plate. The upper part of the cementation device was inserted in the lower part by means of its stamp (Fig. 1A, d), and the whole apparatus was placed in a prosthesis press (RECO Hydromatic Press Typ HMP 1251–4, Reco Dental, Wiesbaden, Germany) (Fig. 1A, e) with pressure buildup (9807 N). Each composite specimen was light-cured with an LED polymerization lamp (Bluephase, Ivoclar Vivadent, Schaan, LIE) from all four sides of the glass plate for 20 s each. Subsequently, the apparatus was removed from the prosthesis press, and the lower side of the specimen was light-cured again for 60 s through the glass plate. By inserting screws in the upper part of the cementation device against the plane surface of metallic stripes in the lower part, the upper and lower parts were separated (Fig. 1A, f), and the composite specimen was removed. The even and smooth bottom side of the composite specimen, which was created by contact with the glass plate under pressure, was used for laser exposure. A cross was scratched with a scalpel into the surface to create four squares (Fig. 1A, g). Only squares free from flaws (e.g., pores), as verified under a light microscope (Olympus SZX7), were used for the determination of the ablation threshold and Er:YAG laser irradiation for laser-induced damage. The specimens were stored in distilled water for 2–4 weeks in an incubator (Memmert INB 500, Memmert GmbH, Schwabach, Germany) at 37 °C.

**Fig. 2:**
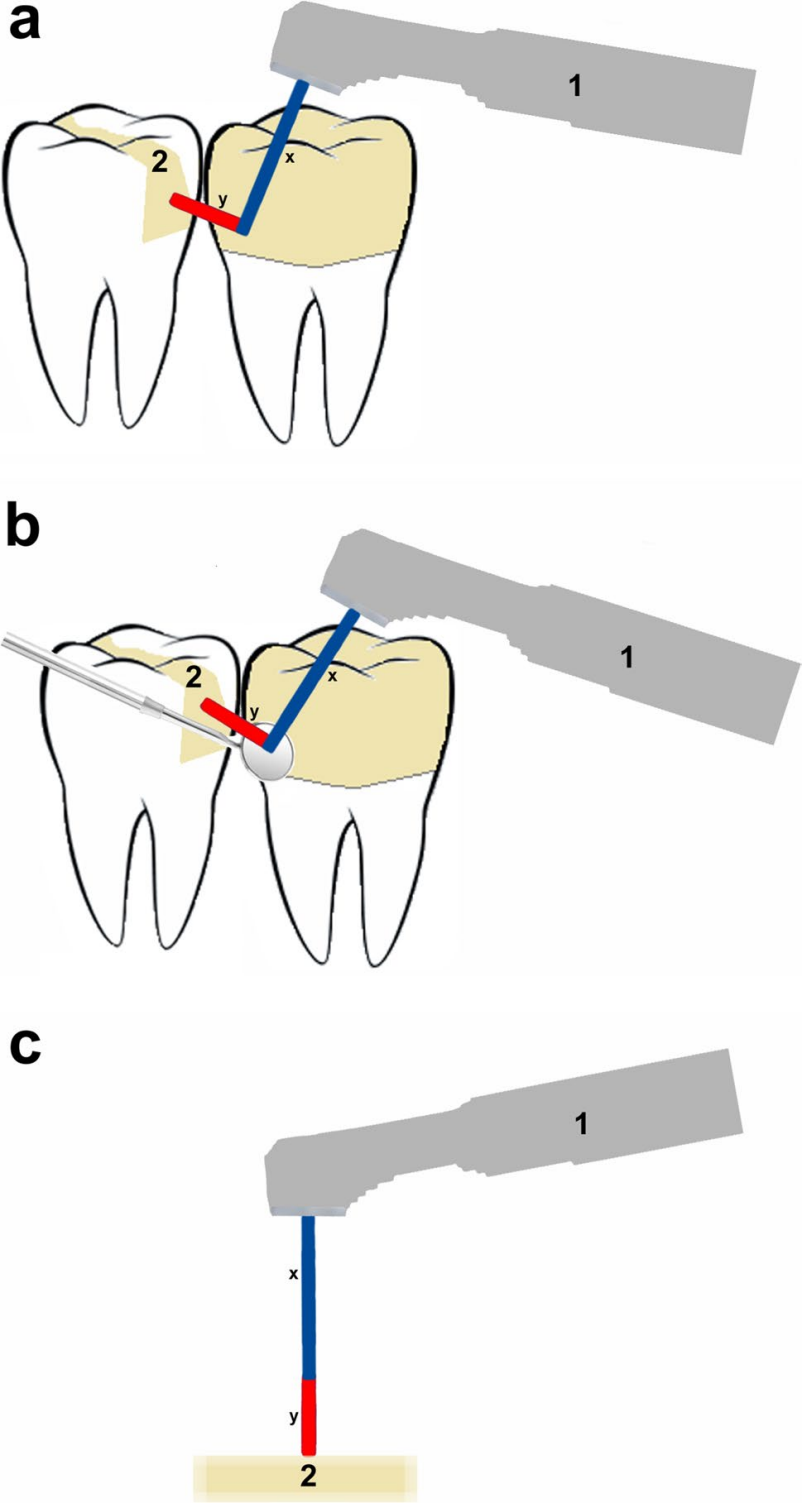
**a** Clinical scenario for indirect inadvertent laser exposure of a composite filling due to reflection at a ceramic crown. **b** Clinical scenario for indirect inadvertent laser exposure of a composite filling due to reflection from a dental mirror. **c** Implementation of the simulation of both clinical scenarios (3a and b) in the in vitro study. x, 10 mm (operating distance of the “direct laser exposure” scenario); y, 5 mm; 1, laser handpiece; 2, nanohybrid composite (as tooth filling in situ in 2a and b and as plane specimen in 2c)

### Ablation threshold

The lowest laser fluence that caused ablation to the composite specimens—the ablation threshold—was determined. The Er:YAG laser beam was perpendicularly directed on a composite specimen square by fixing the laser handpiece and specimen accordingly in an apparatus. An operating distance of 10 mm was chosen, which resulted in the smallest possible laser spot diameter (0.8 mm). The diameter was verified using a test series of laser irradiation with different operating distances on burn paper and subsequent measurement of the laser spot diameter under a light microscope (Zeiss Axiophot, Carl Zeiss AG). The laser was run in the long pulse mode without water and air. The reasons for not using cooling and possible implications for the results are discussed in the final paragraph of the “Discussion” section. The resulting output energy that reached the specimen was measured with an energy meter (OPHIR Laserstar, OPHIR Optronics, Jerusalem, Israel) in units of millijoule and used for the laser fluence calculation (output energy/π × (0.8 mm/2)^2^) in units of Joules per square centimeter. Each specimen square was exposed to a single laser pulse by running the Er:YAG laser with the lowest repetition rate (2 Hz) for 0.5 s. The test series started with a laser fluence of 14.5 J/cm^2^, which was above the ablation threshold and caused damage to the composite square. The test series continued with consecutively lower laser fluences. Subsequently, the specimen was examined under a light microscope (Zeiss Axioplan, Carl Zeiss AG, Oberkochen, Germany). The lowest laser fluence, which barely caused damage to the specimen, was determined as the ablation threshold.

### Er:YAG laser-induced damage for simulated clinical scenarios for inadvertent irradiation

The composite specimen squares were perpendicularly irradiated using the Er:YAG laser beam by fixing the laser handpiece and specimen to an apparatus. Each square was exposed to a single laser pulse by running the Er:YAG laser at the lowest repetition rate (2 Hz) for 0.5 s (long pulse mode). Air and water cooling were not used. The reasons for this and possible implications for the results are discussed in the final paragraph of the “Discussion” section.

Seven irradiation parameters (energy setting and operating distance; Table 1) of the Er:YAG laser were determined as follows. The (1) highest possible energy setting on this Er:YAG laser (KEY laser 3 +) (600 mJ) was chosen to cover the worst-case scenario. In addition, the (2) energy setting for dentin preparation (200 mJ) as recommended by the manufacturer [25] for this Er:YAG laser was chosen to cover a typical dental application of this type of laser. The output energy that reached the specimen was measured with an energy meter (OPHIR Laserstar, OPHIR Optronics, Jerusalem, Israel) in units of millijoule and was used for the laser fluence calculation (output energy/π × (laser spot diameter/2)^2^) in units of Joules per square centimeter. At the (1) highest possible energy setting on this laser, the output energy reaching the specimen was 570 mJ. For the (2) energy setting for dentin preparation, the output energy reaching the specimen was 190 mJ (Table 1). In this in vitro study, these two irradiation parameters simulated inadvertent direct laser exposure, which can occur to immediately adjacent non-target areas, especially in non-contact mode under clinical conditions. Therefore, an operating distance of 10 mm with the smallest possible laser spot diameter (0.8 mm) as derived from the preliminary burn paper tests described above was chosen to achieve the greatest possible laser fluence (worst-case scenario).

**Table 1:** Irradiation parameters at the KEY Laser 3 +

Output energy [mJ]	Operating distance [mm]	Laser fluence [J/cm^2^]	Simulated clinical scenario for inadvertent irradiation
(1) Highest possible energy setting on the KEY laser 3 +			
570	10	113.4	Direct laser exposure
466	15	53.8	Indirect laser exposure: simulated reflection from a dental mirror
57	15	6.6	Indirect laser exposure: simulated reflection from a ceramic surface
(2) Energy setting for dentin preparation as recommended by the manufacturer			
190	10	37.8	Direct laser exposure
155	15	17.9	Indirect laser exposure: simulated reflection from a dental mirror
19	15	2.2	Indirect laser exposure: simulated reflection from a ceramic surface; laser fluence 15% below ablation threshold
(3) Energy setting resulting in subablative laser fluence			
14	15	1.6	Laser fluence 40% below ablation threshold

While working with the (1) highest possible energy setting (600 mJ) on this Er:YAG laser or with the (2) energy setting for dentin preparation (200 mJ), inadvertent reflections may occur on dental instruments or restorations in clinical reality. For this reason, further irradiation parameters were chosen in this in vitro study to simulate inadvertent indirect laser exposure due to reflection from a dental mirror and at a ceramic surface (dental crown) for both energy settings (1) and (2). The two assumed clinical scenarios can be seen in Fig. 2a and b. The laser beam first hits a ceramic crown (Fig. 2a) or a dental mirror (Fig. 2b). It is reflected by this, so that part (see reflection degrees below) of the laser energy hits the composite filling on the adjacent tooth. The total distance (including reflection) that the laser beam travels was set at 15 mm according to the intraoral size ratios. In the present study, these scenarios were implemented as follows (Fig. 2c): the composite specimens were irradiated perpendicularly with the Er:YAG laser as described above for direct laser exposure. However, the operating distance was increased to 15 mm (the laser spot diameter as determined: 1.05 mm), and the output energy reaching the specimens was decreased according to the reflection degrees. These were determined in advance for a dental mirror (Aesculap Dental Mirror DA027R, Braun, Tuttlingen, Germany) and a common ceramic surface (lithium disilicate; IPS e.max CAD HT, color A3, Ivoclar Vivadent, Liechtenstein). For this purpose, these two surfaces were irradiated with KEY Laser 3 + light at an angle of 45°, and the energy of the beam reflected at an angle of 45° was detected using an energy meter (OPHIR Laserstar, OPHIR Optronics, Jerusalem, Israel). To determine the reflection degree, the quotient (in percent) of the reflected energy measured in this way and the energy reaching the mirror’s or crown’s surface was formed and resulted in ~ 80% for the dental mirror and ~ 10% for the ceramic crown. Thus, the input laser energy settings of the Er:YAG laser were chosen, which resulted in accordingly attenuated output energies reaching the specimen compared to direct laser exposure, e.g., 466 mJ (~ 80% of 570 mJ) for simulated reflection from a dental mirror (Table 1). One of the laser parameters for the simulated reflection from the ceramic surface (output energy 19 mJ, operating distance 15 mm) resulted in a laser fluence of 15% below the ablation threshold, as determined above.

**Fig. 3:**
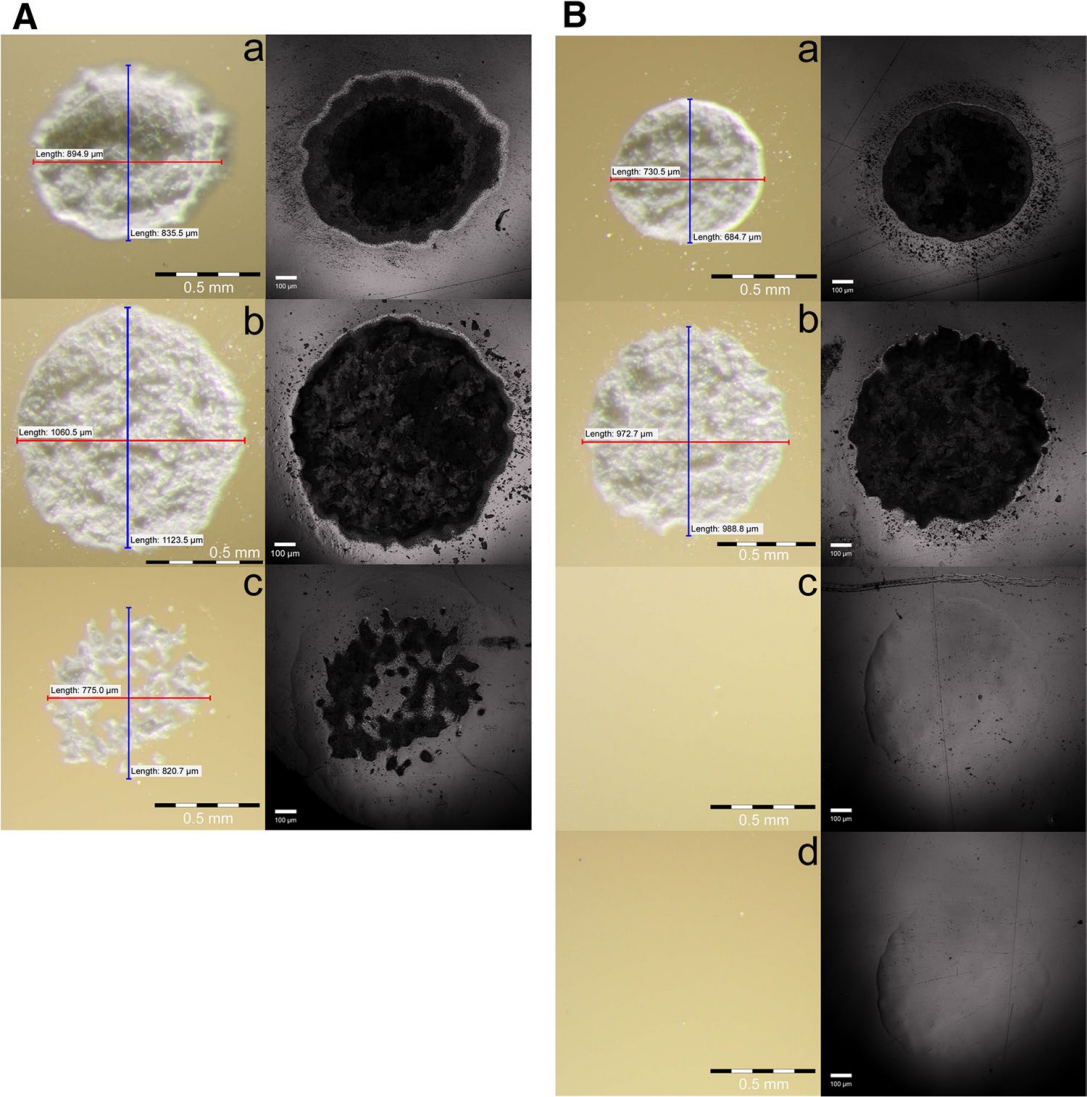
Light microscope (left column) and laser scanning microscope (right column) images of nanohybrid composite specimens (Venus Diamond) after Er:YAG laser exposure with different laser irradiation parameters (OE, output energy; OD, operating distance; LF, laser fluence; SCS, simulated clinical scenario for inadvertent irradiation). **A** (1) Highest possible energy setting on the KEY laser 3 + . a OE: 570 mJ/OD: 10 mm/LF: 113.4 J/cm^2^/SCS: direct laser exposure. b OE: 466 mJ/OD: 15 mm/LF: 53.8 J/cm^2^/SCS: indirect laser exposure: simulated reflection from a dental mirror. c OE: 57 mJ/OD: 15 mm/LF: 6.6 J/cm^2^/SCS: indirect laser exposure: simulated reflection from the ceramic surface. **B** (2) Energy setting for dentin preparation as recommended by the manufacturer and (3) energy setting resulting in subablative laser fluence. a OE: 190 mJ/OD: 10 mm/LF: 37.8 J/cm^2^/SCS: direct laser exposure. b OE: 155 mJ/OD: 15 mm/LF: 17.9 J/cm^2^/SCS: indirect laser exposure: simulated reflection from a dental mirror. c OE: 19 mJ/OD: 15 mm/LF: 2.2 J/cm^2^/SCS: indirect laser exposure: simulated reflection from the ceramic surface; LF 15% below the ablation threshold. d OE: 14 mJ/OD: 15 mm/LF: 1.6 J/cm^2^/SCS: LF 40% below the ablation threshold

Another (3) subablative setting was used (Table 1), resulting in a laser fluence of 40% below the ablation threshold. This was done to evaluate whether thermal effects were still visible.

Er:YAG laser irradiation to produce laser-induced damage was carried out on six composite specimen squares (*n* = 6) for each of the seven irradiation parameters (Table 1).

### Light microscope (LM) measurement (crater diameter)

The laser-induced damage was quantified by measuring the crater diameter with an LM (Olympus SZX7; 3.2-fold magnification; software, Olympus Stream Essentials). An integrated micrometer scale was used to determine the diameter in the horizontal and vertical directions. The mean value of these two measurements was calculated.

### Laser scanning microscope (LSM) measurement (crater diameter/depth and volume)

The same specimens were investigated under an LSM. The laser-induced damage of the specimen was quantified by measuring the diameter of the craters in two directions—horizontally and vertically—and calculating the mean of these two values. With the LSM, the depth and volume of the craters were determined.

The LSM was used with Confocal Microscopy Software in the Expert Mode (Carl Zeiss AG, Release 3.2) with the Objective Plan Neofluar 10 × 0.3. Slices from 1 to 200 µm (1-µm steps) under the surface were captured and used to create 3-dimensional topography models. These models were used to determine the diameters, depths, and volumes of the craters. The diameter was determined on the uppermost layer. The depth was determined by first focusing on the uppermost layer and then on the deepest point of the crater and calculating the difference of these two height settings as given by the software. The ablation volume was determined by fictively filling the crater with simulated liquid.

To check for comparability between the results of the LSM and the standard measuring device (LM) for this application, the intraclass correlation coefficient (ICC) was calculated for the crater diameter for all the ablative laser parameters using a two-way fixed effects model with the unjustified model (“absolute agreement”).

## Results

### Ablation threshold

For the determination of the ablation threshold, six specimen squares were needed. The ablation threshold was determined to be 2.6 J/cm^2^ (Table 2).

**Table 2:** Detection of the ablation threshold

Output energy [mJ]	Operating distance [mm]	Laser fluence [J/cm^2^]	Visible damage (investigation under light microscope)
73	10	14.5	Yes
28	10	5.6	Yes
21	10	4.2	Yes
17	10	3.4	Yes
13	10	2.6	Yes, barely detectable; ablation threshold
12	10	2.4	No

### Light microscope (LM) measurement (crater diameter)

The irradiation parameters resulting in a laser fluence of 2.2 J/cm^2^ and 1.6 J/cm^2^ (Table 1) were subablative (see the ablation threshold above). Under the LM, no effect was visible (Fig. 3).

**Fig. 4:**
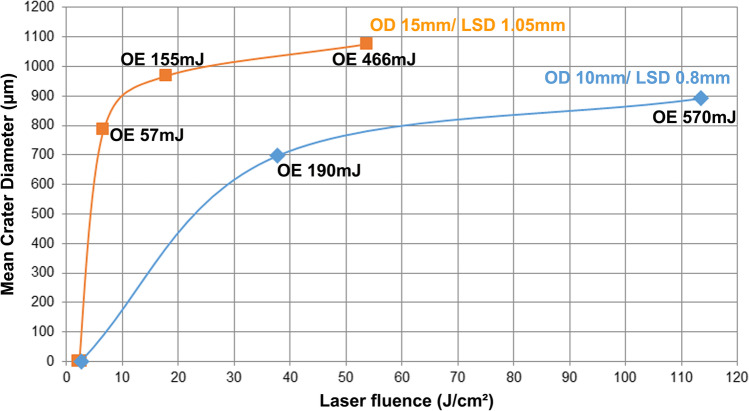
Mean crater diameter in composite specimens after Er:YAG laser exposure as a function of laser fluence with tendency curves; measured with a light microscope (OD, operating distance; LSD, laser spot diameter; OE, output energy)

The five ablative irradiation parameters resulted in craters with varying diameters (Fig. 3). The LM revealed scattered material around the craters for some of these specimens. The lowest ablative parameter, which was used for simulated reflection from the ceramic surface (6.6 J/cm^2^; Table 1), showed no homogenous crater but some interruptions within the crater where the surface was still visible. The crater diameter had the highest value (1075 ± 18 µm) for the simulated reflection from a dental mirror with an operating distance of 15 mm and an output energy of 466 mJ.

The simulated reflection from a dental mirror resulted in a larger crater diameter than direct laser exposure, whereas the simulated reflection from the ceramic surface resulted in an ~ 10% smaller crater diameter for (1) an output energy of 57 mJ and in no crater at all for (2) an output energy of 19 mJ due to the laser fluence being below the ablation threshold (Table 3).

**Table 3:** Crater diameter, depth, and volume measured under a light microscope (LM; only diameter) and under a laser scanning microscope (LSM)

Output energy [mJ]/operating distance [mm]/laser fluence [J/cm^2^] SCS	LM crater diameter [µm] Mean ± SD (Min/Max)	LSM crater diameter [µm] Mean ± SD (Min/Max)	LSM crater depth [µm] Mean ± SD (Min/Max)	LSM crater volume [µm^3^] Mean ± SD (Min/Max)
(1) Highest possible energy setting on the KEY laser 3 +				
570/10/113.4 Direct laser exposure	892 ± 40 (856/965)	907 ± 31 (878/965)	89 ± 2 (86/92)	12,261,667 ± 2,449,591 (10,210,000/16,210,000)
466/15/53.8 Indirect laser exposure: simulated reflection from a dental mirror	1075 ± 18 (1056/1099)	1082 ± 17 (1063/1110)	90 ± 4 (85/95)	11,016,666 ± 711,636 (10,100,000/11,840,000)
57/15/6.6 Indirect laser exposure: simulated reflection from a ceramic surface	785 ± 11 (772/798)	772 ± 21 (733/790)	41 ± 2 (38/43)	3,461,423 ± 257,437 (3,059,452/3,771,813)
(2) Energy setting for dentin preparation as recommended by the manufacturer				
190/10/37.8 Direct laser exposure	698 ± 11 (680/709)	707 ± 12 (687/724)	66 ± 3 (61/69)	7,976,959 ± 664,265 (7,342,383/8,968,877)
155/15/17.9 Indirect laser exposure: simulated reflection from a dental mirror	968 ± 11 (954/980)	973 ± 13 (951/986)	59 ± 4 (55/65)	5,418,006 ± 286,337 (5,088,101/5,883,252)
19/15/2.2 Indirect laser exposure: simulated reflection from a ceramic surface; laser fluence 15% below ablation threshold	No crater; no visible effect	No crater, but melting effect visible		
(3) Energy setting resulting in subablative laser fluence				
14/15/1.6 Laser fluence 40% below ablation threshold	No crater; no visible effect	No crater, but melting effect visible		

*SCS* simulated clinical scenario for inadvertent irradiation; *SD* standard deviation; *Min* minimum; *Max* maximum

**Fig. 5:**
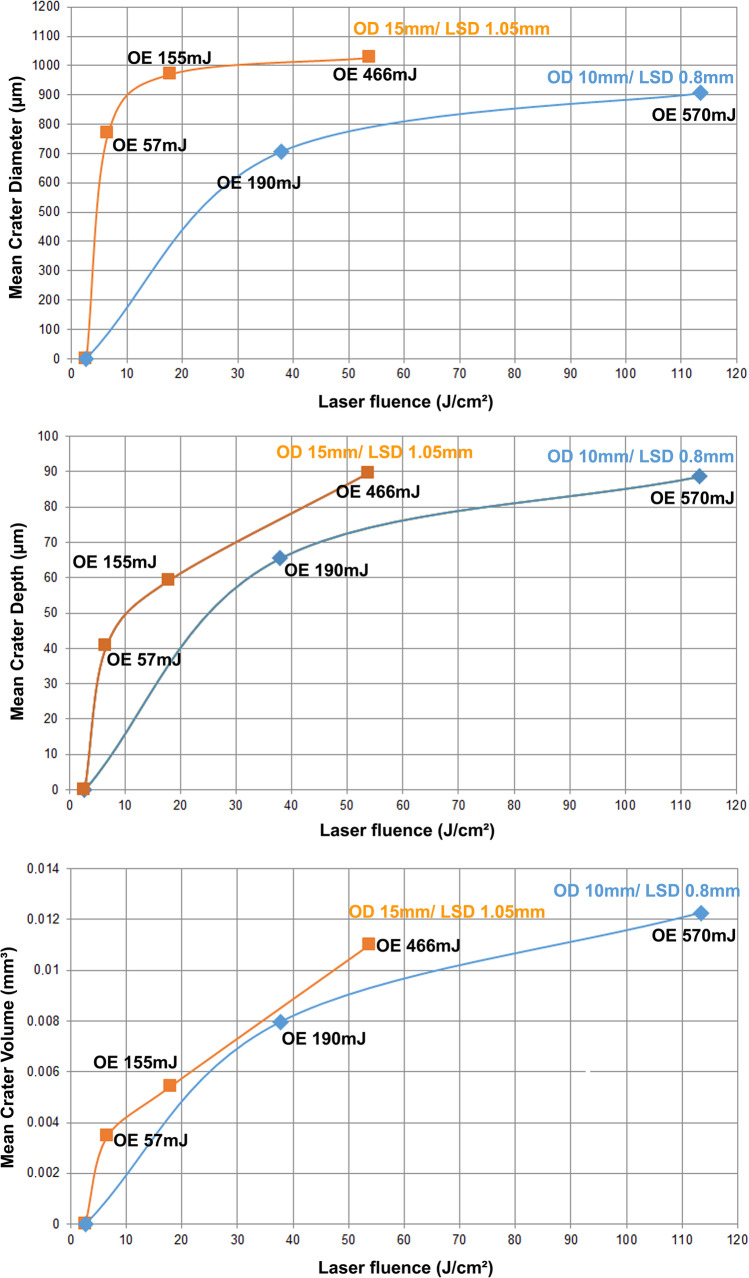
Mean crater diameter, depth, and volume in composite specimens after Er:YAG laser exposure as a function of laser fluence with tendency curves measured with a laser scanning microscope (OD, operating distance; LSD, laser spot diameter; OE, output energy)

The dependency of the mean crater diameter on the operating distance—and thus the laser spot diameter—and the laser output energy is shown in Fig. 4. The two tendency curves show that the larger operating distance of 15 mm (simulated reflection) with the larger laser spot diameter (1.05 mm) resulted in a larger crater diameter for the same laser fluence compared with the smaller operating distance of 10 mm (direct laser exposure) with the smaller laser spot diameter (0.8 mm). The crater diameter increased with increasing laser fluence but tended to reach a saturation value for each operating distance/laser spot diameter.

### Laser scanning microscope measurement (crater diameter/depth and volume)

Using LSM, no crater was visible for the two subablative irradiation parameters. The LSM investigation revealed melting effects (thermal effects) (Fig. 3). The irradiation parameters, which resulted in a laser fluence of 40% below the ablation threshold (Table 1), showed a melting circle, which was 50 µm smaller in mean diameter than the melting circle caused by the other subablative laser fluence (15% below the ablation threshold, Table 1). The lowest ablative laser fluence (6.6 J/cm^2^, Table 1) still showed a continuous melting circle with a larger diameter than the centrally situated crater. Higher laser fluences also resulted in melting effects for almost all specimens; however, the effects were no longer in a continuous circle but were in an interrupted circle. The highest laser fluence (113.4 J/cm^2^, Table 1) showed no melting effects. The LSM investigation revealed scattered material around the craters (Fig. 3) in 6 out of 6 specimens for all laser fluences above 6.6 J/cm^2^ and in 4 out of 6 specimens for a laser fluence of 6.6 J/cm^2^.

In contrast to the dependencies of the crater diameter, as already described above for the LM, the crater depth and volume showed different patterns. The simulated reflection from a dental mirror resulted in an almost equal crater depth for (1) an output energy of 466 mJ (Table 1) and a somewhat smaller crater depth for (2) an output energy of 155 mJ (Table 1) than the respective direct laser exposure. The simulated reflection from a dental mirror resulted in smaller volumes for (1) and (2) than after the respective direct laser exposure. The simulated reflection from the ceramic surface resulted in an ~ 50%/70% lower crater depth/volume for (1) an output energy of 57 mJ than the direct laser exposure and in no crater at all for (2) an output energy of 19 mJ because the laser fluence was below the ablation threshold. The highest crater depth was ~ 90 µm for (1) output energies of 466 mJ (Table 1, simulated reflection from a dental mirror) and 570 mJ (Table 1, direct laser exposure) (Table 3).

The dependency of the mean crater diameter, depth, and volume measured with the LSM on the operating distance—and thus the laser spot diameter—and the laser output energy is shown in Fig. 5. The two tendency curves show that the crater depth and volume are less dependent on the laser spot diameter than the crater diameter, as they are rather closer to each other. The crater volume for the irradiation parameter 190 mJ/10 mm almost touches the 15-mm tendency curve. The crater depth and volume increase with increasing laser fluence, as already described above for the crater diameter. The flattening of the crater depth and volume tendency curves with increasing laser fluence is less pronounced.

The results answer yes to the question of the study, if damage to a nanohybrid composite still occurs with indirect inadvertent Er:YAG laser irradiation when simulating reflection. The damage was of a similar magnitude for most of the clinical scenarios compared to the direct inadvertent irradiation scenarios.

The intraclass correlation coefficient (ICC) for the comparison between the results for the crater diameter measured by the LM versus the LSM revealed very high agreement between both measurement methods (ICC, 0.995; lower/upper limit of 95% confidence interval, 0.988/0.998).

## Discussion

Several clinically relevant Er:YAG laser irradiation parameters, which simulated clinical scenarios for inadvertent irradiation, led to laser-induced damage, in the form of melting and ablation craters, to the nanohybrid composite specimens in this in vitro study. The question of the study was answered as follows: indirect inadvertent Er:YAG laser irradiation when simulating reflection still caused damage to a nanohybrid composite despite the reduced laser fluence. For most clinical scenarios, the damage was even of a similar magnitude compared to the direct inadvertent irradiation scenarios. Concerning the simulated reflection from a common ceramic surface (lithium disilicate) for the lower of both energy setting scenarios (energy setting for dentin preparation), the resulting laser fluence was 15% below the specific composite ablation threshold; thus, no ablation occurred. However, laser scanning microscopy still revealed thermal damage (melting). The same is true for the parameters resulting in a laser fluence 40% below the ablation threshold. These parameters, as given in Table 1, could result from a reflection at a medium with a reflection degree of 7% applying the lower of both energy setting scenarios. For all other clinical scenarios for simulated reflection in this study, both ablation and melting occurred. Even simulated reflection from a common ceramic surface (lithium disilicate) with only ~ 10% output energy reaching the composite specimen still led to craters for the higher of both energy setting scenarios (highest possible energy setting on the KEY laser 3 +). One must keep in mind that we simulated rather worst-case scenarios for reflection, in terms of laser fluence, hitting the composite filling in an immediately adjacent tooth. In the case of more distant non-target fillings (e.g., in the case of missing teeth between the target and non-target), which are hit inadvertently by reflected laser light, the operating distance and thus the laser spot diameter would increase, leading to a lower laser fluence, which might be below the ablation threshold.

For the simulation of different clinical scenarios for inadvertent laser irradiation, we varied the output energy reaching the composite and the operating distance (and thus the laser spot diameter). The crater dimensions depended on both. For the crater depth but especially for the crater volume, the influence of the operating distance was less pronounced. Most studies investigating Er:YAG laser-induced composite ablation work with a fixed laser spot diameter [8, 10–12, 26, 27] and only vary the output energy to change the laser fluence. The present study varied both the output energy and laser spot diameter, resulting in varying laser fluences. The laser fluence alone was not a reliable indicator for the crater dimensions. A higher laser fluence did not necessarily lead to a larger crater diameter or depth. For the same laser spot diameter, a higher laser fluence—and thus output energy—always led to a larger crater diameter, depth, and volume. This increase seemed to be limited, especially for the crater diameter, as the increase was not linear but flattened with higher laser fluence. Other studies applying the Er:YAG laser [11] and a different laser system (CO_2_ laser) [28] also showed this effect [28]. The lower laser fluences resulted in smaller crater diameters than the laser spot diameter, whereas the highest laser fluence for both operating distances resulted in a larger crater diameter than the respective laser spot diameter. Thus, the damage extended beyond the laser spot borders. This extension, however, is very likely to be limited to a saturation value that is not much larger than the specific laser spot diameter. Lizarelli et al. found a saturation value for the crater diameter that was ~ 40% larger than the laser spot diameter for three different types of composites [9].

The crater depth also showed a slight dependency on the laser spot diameter, as the tendency curves show somewhat larger crater depths for the larger laser spot diameter for the same laser fluence. The reason for this may lie in the relationships of the penetration depth of laser light, which depends on the laser wavelength and laser spot diameter [29]. A larger laser spot diameter leads to less lateral scattering in the material, resulting in a greater penetration depth [29]. Consequently, the same laser fluence results in a larger energy input to a given depth for a larger laser spot diameter compared to a smaller laser spot diameter. Thus, for a larger laser spot diameter, the layer up to which the ablation threshold of a material is still exceeded during laser irradiation lies deeper below the surface, resulting in larger crater depths. This phenomenon is a conclusive explanation for the course of the two graphs for the crater depth as described above. Relatedly, it is the plausible reason why the simulated reflection from the dental mirror with less than 50% of the laser fluence, but with an ~ 30% larger laser spot diameter compared to the direct laser exposure scenario, resulted in the same crater depth (for (1) the highest possible energy settings on the KEY laser 3 +) and in an only slightly smaller crater depth (for (2) the lower energy settings).

For the crater depth and volume, a flattening of the curves with higher laser fluence was also recognizable, although less pronounced than for the crater diameter. The same observation was made before in a study analyzing Er:YAG laser-induced craters to three different types of composites [11]. The penetration depth of the laser light depends on the laser wavelength (here, 2.94 µm) and the laser spot diameter as described above [29]. For a constant laser spot diameter, higher laser fluences consequently only lead to more energy input at the same depth, which is very likely to lead to more pulled-out material farther away. This effect, as indicated above, is probably limited to a saturation value that is correspondingly larger for larger laser spot diameters.

The dependency of the crater dimensions on both the operating distance (and thus laser spot diameter) and output energy led to the phenomenon that a simulated reflection with less than half of the laser fluence compared with direct laser exposure did not necessarily lead to a smaller crater in all dimensions. This result is clinically relevant because inadvertent irradiation by reflection is even more likely to stay unnoticed and thus untreated than inadvertent direct irradiation.

The clinical impact of damage in the form of craters depends on the location of inadvertent laser irradiation. Roughening and thus predilection sites for plaque accumulation are caused by the crater dimensions [30]. There is no immediate risk of caries formation in the middle of a filling. At the restoration margin, however, damage to these dimensions has a worse clinical impact. The crater diameters and also the much smaller crater depths are well above the marginal gap widths of poorly adapted fillings [31]. Such leaking restoration margins enhance the risk of secondary caries [32]. If plaque accumulation at a laser-induced crater occurs close to the gingiva, the development of gingivitis and periodontitis is encouraged [33]. The laser-induced roughening may be eliminated by minor grinding and polishing given the dimensions of the crater depth but only if damage is detected.

Data on the frequency of occurrence of this problem in clinical practice are not available even though the problem of laser-induced damage to intraoral structures (here, composite restorations) due to inadvertent direct and indirect laser irradiation due to reflection [23] is repeatedly addressed [2, 24]. More serious laser hazards, such as eye and skin injuries [2], are much more likely to be reported. Even here, laser safety professionals believe that many hazards are not officially reported and thus documented [2].

Two different measurement methods were used for the analysis of the same specimens. The LSM provides a better picture quality and much more information than the LM. However, the standard measuring device LM for this application [8, 10, 21] should be used in addition to checking the comparability of the results of the methods to each other and thus to other studies. The LSM eliminates light scattering due to confocal projection, resulting in enhanced picture quality [34], which is especially relevant for the highly light scattering tooth-colored composite. Only the LSM provided a direct evaluation of all three dimensions, which is crucial for interpreting the clinical impact. With an LM, a three-dimensional evaluation is only indirectly feasible. Next to the crater diameter, the crater depth can be determined with an LM by focusing on the surface and on the deepest point of the crater and calculating the difference [8] or cutting the specimen transversally for a depth measurement [9, 10]. Volume has only indirectly been calculated using an estimated crater form [9, 10, 21]. Because the crater conical shape varies to a certain extent, this method, however, has clear limitations in precision. Another advantage of LSM is that it provides more detailed imaging. Only the LSM revealed melting effects, and it also showed scattered material around the craters more reliably. However, when focusing only on the crater diameter, the LM was the more convenient measurement method and was easier to apply. The analysis of comparability showed that the LSM provided comparable results to those of the standard measuring device LM. Thus, a comparability between studies for the determination of the crater diameter with these two measuring devices is given. Both procedures are nondestructive methods without complex specimen preparation after irradiation. Because of the additional information and comparability of results for the crater diameter, the use of the LSM rather than the LM for analyzing ablation craters seems advisable in future studies.

The melting effects revealed by the LSM are in accordance with the mechanism of ablation described for composites. Here, rapid melting occurs upon Er:YAG laser irradiation [8], resulting in large expansion forces due to the volume increase upon melting and thermal vaporization [7, 11]. This results in explosive ejection of material and thus ablation craters [8, 11]. Evidence of these melting processes has only occasionally been reported, as in a study applying a scanning electron microscope for composite crater evaluation [7]. In another study using an LM, only small signs of melting could be detected [8]. In the in vitro study at hand, only the LSM, but not the LM, revealed melting effects. Thus, it seems possible that melting effects were not detected or underestimated in studies that only used LM.

Due to the additional information gained by the LSM about the melting effects, the following interpretation is possible. For the two subablative parameters (laser fluence: 1.6 J/cm^2^ and 2.2 J/cm^2^, see Table 1), the energy input was sufficiently high to melt the material but too low to ablate it. For the ablative parameters (laser fluence: 6.6 J/cm^2^, 17.9 J/cm^2^, 37.8 J/cm^2^, 53.8 J/cm^2^, 113.4 J/cm^2^, see Table 1), the radially decreasing energy due to the non-tophead beam profile of this laser type resulted in a sufficiently high energy input above the specific ablation threshold to cause ablation only in the center analogous to the processes in enamel and dentin [19]. For dental hard tissue, the underlying main mechanism for ablation differs, but when the ablation threshold is exceeded, material is also explosively torn out, and craters are formed as well [19]. In the case of composite, the energy input, although insufficiently high in the periphery to ablate the material, was still able to melt it. With cooling, the melted material solidified again, resulting in melting circles around the crater, as shown in the LSM images. The melting effects were less pronounced with higher laser fluences and were no longer detectable for the highest laser fluence. The most likely explanation is that the composite particles were ejected more intensively for higher laser fluences, carrying peripheral areas away, which would have shown melting effects otherwise.

The ablation threshold of 2.6 J/cm^2^ determined in this study is in the lower range of the Er:YAG laser ablation thresholds (2.6–4.7 J/cm^2^) determined for four other representatives of the composite material class [17]. Compared with the range of ablation thresholds determined for dentin (2.7–4 J/cm^2^) [15, 16], it is only minimally lower. Other representatives of the composite material class with somewhat higher ablation thresholds reach the ablation threshold of dentin accordingly [17]. Compared with the range of ablation thresholds determined for enamel (3.9–11 J/cm^2^) [13–15], the ablation thresholds of the composite used in this study and of another representative of this class of material [17] are lower. However, the laser fluences, which were applied in the clinical scenarios simulated here for inadvertent laser irradiation, were also well above the range of ablation thresholds for enamel in the majority of cases. Therefore, ablation of dentin and enamel can also be assumed when simulating the scenarios of this in vitro study with the applied laser parameters for dental hard tissue.

The depths of the craters are in the same range as the calculated average ablation rates per pulse by Wigdor et al., which was up to 80 µm for composites with laser fluences of up to 90 J/cm^2^ [12] when applying a comparable experimental setup. Compared with the ablation rate of a single Er:YAG laser pulse on dentin, the resulting crater depths determined in this in vitro study in composite show approximately the same values as in dentin (~ 62 µm) for a laser fluence of 30 J/cm^2^ [19]. The crater depth in enamel is comparable to that in dentin for the first few pulses [19]. This finding fits with the statement of Hibst et al. that the crater depths per pulse for the composites investigated were comparable to those measured for enamel and dentin [8]. One must keep in mind that comparisons of the crater dimensions to other studies are often difficult to perform due to multiple factors, such as the laser settings used. For instance, most studies applied multiple laser pulses instead of only one.

The composition and age of the composite also influence the Er:YAG laser ablation efficiency [10, 11]. In a comparative study investigating the Er:YAG laser ablation of three different composites, a hybrid composite could be ablated more easily than a microfilled and condensable composite [11]. The authors speculated that the reason may be that the resistance against the material ablation relies entirely on the polymeric matrix due to the microstructure of the hybrid composite. Because of this, it is easier to ablate than the condensable composite in that study, where the material cohesion seems to be reinforced by the heterogeneous structure containing fiberglass [11]. However, the same group of authors also showed that the ablation rates of the three composite classes were in a similar range. Compared to enamel, for example, the ablation rate was always several times lower [9]. Next to the composition, the age of the composite also influences the Er:YAG laser ablation efficiency. Lizarelli et al. stated that aged composite specimens are more easily ablated than recently cured, non-aged composites and indicated the more fragile surface characteristics of aged specimens as a probable reason [10]. We used artificially aged, nanohybrid composite specimens in this in vitro study. Due to the results of other studies, as mentioned above, we may speculate that our composite specimens belong to the group of those, which are somewhat easier to ablate.

We applied single laser pulses, resulting in “single pulse craters.” Higher repetition rates consequently lead to the application of several laser pulses. If the handpiece or the reflector (e.g., dental mirror) is moved during inadvertent irradiation, a series of side-by-side “single pulse craters” result. If the laser beam stays fixed during inadvertent irradiation, the same spot will be hit several times. As the absorber is progressively consumed, the ablation threshold is likely to increase and the laser parameters may no longer be sufficiently high to ablate the material, which brings the thermal effects to the fore, possibly leading to more melting, as shown before [7]. The present study provides information about the minimal crater dimensions caused by a single laser pulse, which already shows a clinically relevant extent.

The laser irradiation experiments were performed on six composite specimen squares for each laser irradiation scenario. In another study on Er:YAG laser-induced ablation, nine [12] or 15 [9] specimen spots were irradiated for each group. The comparatively low number of repetitions performed here may cause bias in the results. However, the standard deviations for the crater diameter and depth are rather low for all groups, indicating only small deviations between the repetitions.

Reflection was simulated by reducing the output energy and enhancing the operating distance, resulting in a higher laser spot diameter. Otherwise, we used the same experimental setup as used for direct laser exposure, resulting in a circular laser spot. This scenario corresponds to reflection under one specific angle. However, reflection under a different angle leads to elliptic laser spot formation and thus higher laser spot areas. A larger crater diameter is likely to result given a laser fluence above the ablation threshold. We chose this setup with the smallest possible laser spot diameter for reflection to simulate the worst-case scenario concerning laser fluence.

No air or water cooling was used during our experiments. As the ablation of dental materials is associated with greater thermal side effects than the ablation of natural dental hard tissues [8], working on dental materials without cooling is particularly harmful (worst-case scenario). This scenario occurs in routine clinical practice if cooling is switched off accidentally, if the target is shielded by cavity walls from the cooling agent or in case of inadvertent indirect irradiation by reflection. The influence of water cooling on the extent of composite ablation is complex and depends on the water flow rate [10, 12] and, most likely, on the specific composite, leading to contradictory conclusions in the literature, i.e., water enhances composite ablation [10] and only has a small effect on it [12].

## Conclusion

Inadvertent laser irradiation can occur directly to immediately adjacent non-target tissue or indirectly to more distant non-target tissues due to reflection. Reflected light shows attenuated energy and travels a longer distance, usually resulting in a larger laser spot diameter. Both effects result in a lower laser fluence. The simulated scenarios for Er:YAG laser reflection in this in vitro study still caused damage to a nanohybrid composite. The damage was even of a similar magnitude compared to the direct inadvertent irradiation for most scenarios despite the lower laser fluence. This results from the fact that the larger laser spot diameter proved to have an enlarging effect on the ablation crater diameter and depth. The three-dimensional extent of the craters caused roughening, which may lead to plaque accumulation under clinical conditions. Depending on the site of damage, secondary caries, gingivitis, and periodontitis may thus be instigated if the laser-induced damage remains unnoticed and goes untreated. Clinicians should be aware that reflected laser light is often still able to create such craters in composites. This is true for aged, nanohybrid composite specimens—as investigated here—and may be different for other composites to some extent. Clinical consequences may consist of abandoning highly reflective media such as the usual dental mirror. Since reflection cannot be completely avoided (e.g., on the target tissue itself), a final check using dental optical magnification devices should not be limited to the immediately adjacent tissue but also seems advisable in somewhat more distant areas (e.g., neighboring teeth).

Another conclusion from the present in vitro study for researchers in this field lies in the findings from the comparison between a laser scanning microscope LSM and the standard measuring device—a light microscope LM—for detecting and quantifying laser-induced damage. The LSM provided more information than the LM, including melting effects and scattered material around the craters in a more reliable way. It also showed comparable results to the results of the LM for the crater diameter. For all these reasons, the use of the LSM rather than the LM for analyzing ablation craters seems advisable in future studies.
